# Intestinal Membrane Function in Inflammatory Bowel Disease

**DOI:** 10.3390/pharmaceutics16010029

**Published:** 2023-12-25

**Authors:** Daisuke Nakai, Masateru Miyake

**Affiliations:** 1 Drug Metabolism & Pharmacokinetics Research Laboratory, Daiichi Sankyo Co., Ltd., 1-2-58 Hiromachi, Shinagawa-ku, Tokyo 140-8710, Japan; 2 Pharmapack Co., Ltd., 1-27 Nakaokubo, Toyama 939-2243, Japan; ma-miyake@hanshin-group.co.jp

**Keywords:** inflammatory bowel disease, ulcerative colitis, Crohn’s disease, Ussing chamber, permeability, electrophysiological parameter

## Abstract

Inflammatory bowel disease is a set of chronic inflammatory diseases that mainly develop in the gastrointestinal mucosa, including ulcerative colitis and Crohn’s disease. Gastrointestinal membrane permeability is an important factor influencing the pharmacological effects of pharmaceuticals administered orally for treating inflammatory bowel disease and other diseases. Understanding the presence or absence of changes in pharmacokinetic properties under a disease state facilitates effective pharmacotherapy. In this paper, we reviewed the gastrointestinal membrane function in ulcerative colitis and Crohn’s disease from the perspective of in vitro membrane permeability and electrophysiological parameters. Information on in vivo permeability in humans is summarized. We also overviewed the inflammatory bowel disease research using gut-on-a-chip, in which some advances have recently been achieved. It is expected that these findings will be exploited for the development of therapeutic drugs for inflammatory bowel disease and the optimization of treatment options and regimens.

## 1. Introduction

The intestinal mucosa plays an important role in absorbing nutrients such as glucose, amino acids, and vitamins [1]. In addition, the lumen of the intestinal tract contains a large number of foreign substances, to which the surface of the intestinal epithelium is constantly exposed. Specifically, in the duodenum and upper small intestine, there are many dietary antigens, while the lower small intestine and large intestine harbor numerous gut bacteria and bacterial toxins such as endotoxins. Therefore, the intestinal epithelium plays an important role as a barrier to control the invasion of these inflammatory foreign substances, while also being responsible for the digestion and absorption of nutrients and functional components [1]. Namely, the intestinal tract separates the inside and outside of the body via a barrier layer consisting of intestinal epithelial cells. Meanwhile, the intestinal immune system allows the entry of beneficial substances and the elimination of detrimental ones. When this intestinal epithelial barrier is compromised, foreign substances not only invade the mucosa and cause local intestinal inflammation triggered by the breakdown of the intestinal immune system, but they can also reach distant tissues through the bloodstream, leading to systemic inflammation and contributing to the onset and progression of various diseases [2]. In fact, the diseases characterized by intestinal barrier dysfunction include inflammatory bowel diseases (IBDs), irritable bowel syndrome, and colorectal cancer, as well as non-digestive disorders such as obesity, liver diseases, and skin conditions [3,4,5,6]. From this perspective, the intestinal epithelial barrier has attracted attention as a target for the treatment and prevention of diseases [7,8].

Among the diseases mentioned above, IBD is one of the contexts in which barrier function has been most intensively researched. IBD is a collective term for a group of inflammatory diseases of the gut, including ulcerative colitis (UC) and Crohn’s disease (CD), and it is a chronic inflammatory disease of unknown cause [9,10]. It was originally described as a disease that more commonly affects Caucasians, but now, patients are rapidly increasing in Asia, including Japan, although there are limited epidemiological data from developing countries [11,12,13,14]. In terms of the site of involvement, UC is specific to the colon, while CD is not specific to a particular intestinal region, being observed at sites throughout the digestive tract [15,16].

The importance of the microbiome in the pathogenesis of IBD has increasingly been shown. Indeed, recent studies have revealed the critical role of the gut microbiome in the pathogenesis of IBD and its impact on gut health [17]. Recent clinical and experimental evidence supports the concept of the gut–brain axis, highlighting the interaction between the central nervous system and the gut microbiota, which is closely related to the bidirectional interactions of inflammatory bowel disease and central nervous system disorders [18,19].

**Figure 1:**
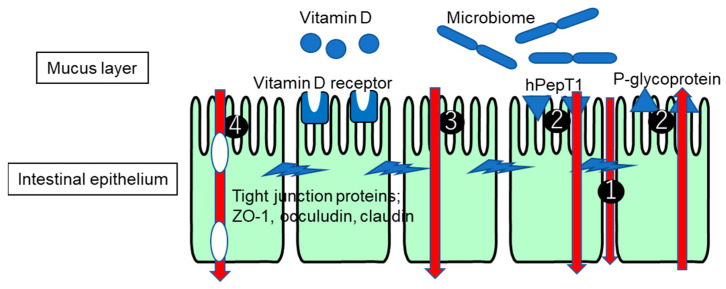
Schematic illustrations of representative players involved in intestinal membrane barrier function and passage route across intestinal epithelium.

This review summarizes intestinal barrier function, including the microbiome involvement (Figure 1), and further focuses on a comparison of the barrier functions of tight junction proteins and the membrane permeability between patients with UC or CD and healthy subjects. 

1: Paracellular route of pore pathway and leakage pathway for larger hydrophilic molecules.

2: Transporter-mediated influx via PepT1, etc., and efflux via p-glycoprotein, etc.

3: Transcellular route for small lipophilic and hydrophilic molecules.

4: Endocytotic route (vesicles shown as white oval), followed by transcytotic route and exocytotic route, for larger peptides and proteins.

The interplay of molecules, not limited to those shown in Figure 1, contributes to the maintenance and destruction of the intestinal membrane barrier function related to the pathogenesis of IBD. Tight junction proteinssuch as ZO-1, occuludin, and claudin are involved in barrier function and are regulated by extracellular signals such as those caused by vitamin D. Vitamin D, via binding to the vitamin D receptor, exerts the suppression of inflammation and the strengthening of the intestinal barrier through the direct suppression of B and T cell proliferation and the regulation of cytokines. The microbiome is suggested to be involved in p-glycoprotein expression, whose decrease causes colonic inflammation. The expression of PepT1, which transports bacterial peptides, is changed in UC and CD patients. 

## 2. Permeability through Epithelial Cells 

There are several routes for solutes to pass through the intestinal epithelium. Such routes depend on the size, hydrophilicity, and transporter/receptor recognition of the solutes. The transcellular route for small hydrophilic and lipophilic compounds and the paracellular route of ions, water, and larger hydrophilic compounds are relevant routes, considering the physiology of the intestinal epithelium. Other routes include the transporter-mediated influx and efflux of nutrients such as sugars, amino acids, and vitamins, as well as xenobiotics, and the endocytotic route for larger peptides and proteins [20,21]. Changes in membrane permeability have been considered important to understanding the intestinal status of UC and CD patients [22].

## 3. Intestinal Tight Junction

Tight junction (TJ) structures expressed in intestinal epithelial cells are important constituents of this barrier, creating a physical obstacle that prevents xenobiotics from crossing the intercellular space. TJ structures are adhesion apparatuses composed of multiple molecules such as occludin, claudin, and zonula occludens (ZO), and are known to be dynamically regulated by extracellular signals [23,24,25].

At least two modes of paracellular pathways are known to be regulated by TJs. One is called the “pore pathway”, which allows small molecules such as electrolytes with a diameter of 0.6 nm or less to pass through in a charge-selective manner [26]. The other is called the “leakage pathway”, which allows a very small number of molecules with diameters up to ~12.5 nm to traverse the epithelial barrier, regardless of their charge [26]. 

The subtypes of claudins that make up TJ strands include channel-type claudins that form small, cation-selective channels in the extracellular part of TJs [26,27,28,29]. Channel-type claudins are highly expressed in specific epithelia, and achieve physiological epithelial transport by charge-selectively permeating electrolytes and coupling with transcellular pathway transport. This channel-type claudin-mediated electrolyte permeation route in the intercellular space corresponds to the pore route described above. Immune signals such as IL-13 and IL-22 can induce the transcription and expression of intestinal epithelial claudin-2, leading to increased permeability via the pore pathway [30,31,32]. Meanwhile, the mechanism behind the leakage route is still poorly understood [33]. One possibility is that, when TJ strands repeatedly break and reconnect, a small number of molecules gradually pass through the intercellular space during the periods of breakage [34]. This permeability can be regulated by long myosin light chain kinase splice variant 1 [35,36]. Under physiological conditions, claudins are expressed in a regionally and locationally specific manner in the gut [37]. Additionally, claudin gene expression varies within intestinal tissues. For example, in the colon, ion pore-forming leaky claudins are restricted to the crypt base [38], whereas tight-sealing claudins accumulate near the surface of the lumen [38]. Current theories on the development of IBD hold that inflammation increases gut leakiness, in part through alterations in claudin expression and, therefore, barrier function [38,39].

The barrier-forming claudins include claudin-1, -3, -4, -5, -6, -8, -9, -11, -14, and -18 for cations and claudin-7 and -19 for anions [27,38,39], while leaky pore-forming claudin-2 and -15 were identified to contribute to increased paracellular permeability for water and (inorganic) cations [23,27,40].

Several studies have reported differential effects of such inflammatory mediators on TJ proteins. The increased expression of claudin-1, -2, and -18 [41,42,43,44] and the downregulation of claudin-3, -4, and -7 were reported in ulcerative colitis [41,43]. Meanwhile, in Crohn’s disease, the upregulation of claudin-1 and -2 and the downregulation of intestinal epithelial claudin-3, -5, -8, and -12 were observed [41,45,46,47], which are associated with changes in the tight junction structure and marked barrier dysfunction [45]. This leakiness increases the probability that gut antigens will penetrate into the body, further perpetuate inflammation, and exacerbate disease. RNA-seq studies of UC and CD patient biopsies have revealed altered claudin levels, with increases in claudin-1, -2, and -18 and decreases in claudin-3, -4, -5, -7, -8, and -12 [38,39].

On the cytoplasmic side of TJs, there are lining proteins such as ZO-family proteins consisting of ZO-1, ZO-2, and ZO-3, which directly bind to the membrane proteins [48,49]. ZO-1 and ZO-2 directly bind to the cytoplasmic domain of claudins and are essential for TJ strand formation. In UC and CD, a reduced expression of ZO-1 was reported, along with an increased expression of claudin-2. The combination of changes in claudin-2 and ZO-1 may result in the loss of selective permeability in patients with UC and CD [50].

The accepted model for the mechanism of the formation of tight junctions to date has been that a cell adhesion molecule (a claudin) binds to the lining protein ZO, gathers it on the cell membrane, and forms a tight junction. This model is based on an analogy with the mechanism of the formation of a different cell adhesion device, the adherens junction [51]. However, whether this model of how tight junctions form is valid has not been confirmed. Very recently, Shigetomi et al. [52] discovered that claudin accumulation, which is necessary for the formation of tight junctions, relies on the interaction of claudins with cholesterol-rich membrane domains, rather than binding with the lining protein ZO. In cells in which claudin expression has been completely abolished, even when claudin mutants that do not bind to ZO proteins are expressed, they still have the activity of forming tight junctions, but they lack palmitoyl, which is necessary for interaction with cholesterol. It has been revealed that claudins with a mutated oxidation modification are unable to form tight junctions, even though they retain the ability to bind to ZO proteins. Further investigations are required to elucidate the interactions between tight junction molecules in conjunction with IBD.

## 4. Changes in Expression of Transporters and Enzymes under IBD

Changes in the expression of transporters under inflammatory conditions have been reported [53]. Within this topic, the focus has mainly been placed on the efflux transporters of the p-glycoprotein and breast cancer resistance protein (BCRP), which have important roles in maintaining the barrier function of the gut mucosa. The protein expression levels of these transporters, as determined by Western blotting, were found to be strongly reduced during active inflammation in patients with UC [54]. Moreover, an immunohistochemical analysis demonstrated a reduced expression of both of these transporters, but only in the inflamed tissue of patients with active UC [55]. It was also reported that, in UC patients, the gene expression of the transporters of ABCB1 (p-glycoprotein), ABCG2 (BCRP), and monocarboxylate transporter 1 (MCT1, SLC16A1) was significantly decreased during inflammation. In contrast, the multidrug resistance-protein 4 (MRP4, ABCC4), organic anion-transporting polypeptide 2B1 (OATP2B1, SLCO2B1), and organic cation transporter-like 2 (ORCTL2, SLC22A18) were significantly elevated in inflamed tissue. However, at the protein level, these findings could only be confirmed for MCT1 [56]. A recent targeted proteomic analysis using liquid chromatography with tandem mass spectrometry provided a more quantitative examination with observations of significant decreases in the p-glycoprotein, MRP4, MCT1, and the enzymes CYP3A5 and UGT2B7 in the inflamed tissues of UC. The expression levels of other proteins such as OATP2B1, CYP3A4, CYP2B6, and UGT2B15 were unchanged during inflammation. BCRP expression was detected only in 4 out of 71 biopsies [57]. The expression of the di-/tripeptide transporter of PepT1 was reported to be increased in UC and CD patients, even though little expression of PepT1 was observed in healthy colons [58,59], while the PepT1 protein was not detected, even in inflamed regions [56,57]. These results are controversial in part; however, the altered transport and metabolism of xenobiotics in the colon of UC patients during active inflammation potentially suggests variability in the disposition of drugs in patients, which would affect the treatment outcome. 

Limited information has been revealed on the relationship between transporter expression and IBD treatment. 5-Aminosalicylic acid (5-ASA) has been widely used as a representative drug for the treatment of IBD [60,61]. 5-ASA at a clinically relevant concentration was reported to have inhibitory potential for PepT1-mediated transport [62]. Both an in vitro human model and an in vivo animal model suggested that the PEPT1-mediated uptake of bacterial peptides such as formyl-methionine-leucine-phenylalanine stimulates the expression of  major histocompatibility complex class 1 molecules, leading to an increased sensitivity to antigen presentation to upregulate inflammatory responses [58,63]. Combined with the finding of the upregulated expression of PepT1 in IBD patients, it was suggested that 5-ASA contributes to the treatment of IBD by decreasing the uptake of bacterial peptides via PepT1 inhibition. It has been reported that 5-ASA is a substrate for OATP, including OATP2B1 [64]. Therefore, it is suggested that changes in the transport function of OATP may affect the therapeutic efficacy of 5-ASA. However, it has been reported that there is no difference in the 5-ASA treatment response based on a single-nucleotide mutation of OATP, suggesting that there are factors determining the therapeutic efficacy that cannot be explained solely by transport function [65].

## 5. Intestinal Membrane Permeability and Electrophysiological Parameters

### 5.1. In Vitro Membrane Permeability

An alteration to the permeability of the gastrointestinal mucosa has been reported not only in gastrointestinal diseases, such as inflammatory bowel disease, functional dyspepsia, and irritable bowel syndrome, but also in non-digestive diseases, such as diabetes and pollen allergies [2,3,4,5,6]. An evaluation of the mucosal membrane permeability of tissue specimens collected under an endoscope using Ussing chamber experiments would help us elucidate the pathogenesis and its molecular mechanism. The Ussing chamber system is a useful experimental option for comparing the gut integrity between healthy and disease states in both animal models and humans from the perspective of membrane permeability [66,67,68]. The main advantages of the application to human intestinal tissue are the maintenance of the morphological structure and the functional expression of ion channels and transporters that reflect in vivo conditions. The presence of those proteins, as well as the mucous layer adjacent to the villus tip of enterocytes, provides a more comprehensive picture regarding the passage of molecules and electrophysiological parameters. However, reports on functional assays of the membrane permeability in UC and CD patients are limited [69]. Katinios et al. showed that UC patients in remission and irritable bowel syndrome (IBS) patients had a reduced TEER and an increased paracellular passage of ^51^Cr-EDTA [69]. Furthermore, ulcerative colitis patients, even during remission, demonstrated a leakier barrier than IBS patients.

Nakai et al. [70] examined the transport characteristics in the intestinal tissues of severe UC patients in a mini-Ussing chamber system using three types of model drugs: fluorescein isothiocyanate (FITC)-dextran 4, a very low-permeability marker via the paracellular route; rebamipide, a low-permeability marker of a p-glycoprotein substrate [71]; and metoprolol, a high-permeability marker via the transcellular route. It was demonstrated that, in UC patients, there were no remarkable changes in the transport index, which is an index of the sum of permeated and tissue-accumulated molecules, with the consistency of absorption rank order. Interestingly, permeated molecules and tissue-accumulated molecules in tissues with severe fibrosis showed a decrease and an increase, respectively, for every tested compound. This suggests that it would not be necessary to administer a different medication depending on the severity of the disease. The unchanged permeability of rebamipide, a p-glycoprotein substrate, was in accordance with the finding in a previous study, but not in agreement with another finding. Although the reasons for this discrepancy are unclear, it might be due to different populations being included in each study. The simultaneous determination of transporter expression and membrane permeability would make it possible to understand their relationships more precisely. The paracellular route markers ^51^Cr-EDTA and FITC-dextran 4 showed different changes in permeability in tissues from UC patients. The molecular weights of ^51^Cr-EDTA and FITC-dextran 4 are 348 and 4000, respectively. The effect of the disease state on the paracellular permeability through the pore pathway is suggested to be dependent on the molecular weight.

In CD patients, the ^51^Cr-EDTA membrane permeability was increased in inflamed tissue compared with that in uninflamed tissue from matched patients [72]. The ^51^Cr-EDTA membrane permeability in the uninflamed tissue was almost equal to that in the tissues from CD patients without an inflamed area. Recently, the ^51^Cr-EDTA membrane permeability was suggested to be related with the accumulation of enteric glial cells (EGC), which are known to be regulators of gastrointestinal functions. Moreover, increased paracellular permeability from EGC mediators was observed in CD patients, whereas this permeability was decreased by these mediators in controls [73].

An examination of the permeability in IBD patients after treatment for the disease would provide novel insights for understanding the mechanism of action of treatment. Infliximab is a neutralizing antibody for tumor necrosis factor alpha to treat CD. The colonic passage of colon-specific adherent-invasive *Escherichia coli* HM427 and the ^51^Cr-EDTA permeability were increased in CD patients, but an infliximab treatment restored both to their control levels [74].

### 5.2. Electrophysiological Parameters

It has already been reported that the electrophysiological parameters of the potential difference (PD) and resistance (TEER) in UC patients appear to decrease in a histological-grading-dependent manner [75]. Physiological parameters of the membrane capacitance were examined in different tissues of the ascending colon and sigmoid colon from UC patients [76]. Furthermore, a deficiency in the epithelial barrier, determined by the current impedance, was characterized and quantified using electrophysiological imaging and a current impedance analysis [75,77].

In more severe cases of both UC and CD, as revealed by determining the extent of ulceration and mucosal thickening at autopsy, there were lower values of PD and R than in normal tissues, although the short-circuit current (Isc) values were not significantly different, irrespective of autopsy grade [78]. In addition, larger changes in the R values in UC tissues than in CD tissues were observed in comparison with control tissues. Functionally impaired active ion transport via ion pumps was suggested by the reduced PD. Irrespective of the observations of changes in the electrophysiological parameters, the permeability of FITC-dextran with a molecular weight of 4 kDa was almost equal among the control, UC, and CD patients. Another study [79] demonstrated that the decreased transepithelial resistance in active UC was restored in remission to the same range as in controls. The paracellular permeabilities for fluorescein, with a molecular weight of 332 Da, and FITC-dextran, with a molecular weight of 4 kDa, aligned with the results regarding resistance. Additionally, these trends in R and the membrane permeability paralleled the expression of tricellulin [79], a tricellular tight junction protein, which is responsible for preventing macromolecule transport via the paracellular route [80]. Similar results were also observed in IBS patients [81].

Meanwhile, the TEER values in CD patients vary among published reports. A significantly lower R was observed in biopsies from CD patients with active disease than in control subjects without a significant change in FD-4 permeability [82]. R in the colon of CD patients with mild or moderate inflammation did not change between patients with active and inactive disease in a remission state when compared with healthy subjects. In contrast, the epithelial resistance, assessed using a transmural impedance analysis, was decreased only in tissues from patients with active CD [45]. This suggests the importance of a current impedance analysis in addition to measurements of the conventional short-circuit current in an Ussing chamber. Collectively, these results suggest that the effect of UC on barrier function is consistent among the published reports, while the reported outcomes of the electrophysiological parameters in CD patients vary.

The electrophysiological and membrane permeability data described here can contribute to a better understanding of the association with altered pathways via paracellular and transcellular routes in IBD patients. However, conflicting results on the electrophysiological parameters have been reported. Further studies are required to elucidate what changes occur in IBD patients under the same experimental conditions.

### 5.3. In Vivo Sugar Test in Humans

Membrane permeability has long been evaluated in IBD patients by administering polyethylene glycol 400 or ^51^Cr-EDTA, or by performing a sugar test using a cocktail of lactulose, rhamnose, and mannitol. Because they have different molecular weights and are less susceptible to metabolism after absorption, their urinary excretion rate is measured and used as an indicator of membrane permeability. Lactulose is a large oligosaccharide that does not usually undergo paracellular transport and can be adsorbed only when there is leakiness of intercellular junctions; meanwhile, mannitol is a smaller molecule that can freely cross the intestinal epithelium. In sugar tests, the sucrose excretion rate, the ratios of lactulose/mannitol or lactulose/rhamnose (L/M or L/R, respectively) excretion rates, the sucralose excretion rate, and the ratio of sucralose/mannitol excretion rates are used as indicators of gastric permeability, intestinal permeability, colonic permeability, and whole-gut permeability, respectively. Representative results of these are as follows.

It has been reported that the ^51^Cr-EDTA permeability remains unchanged in UC and increases in celiac disease [83]. It has also been shown that there is no difference in the membrane permeability among different L/M and L/R ratios in UC and CD patients [84]. In another study, the L/M ratio was found to not differ among IBS, UC, and healthy control patients [85]. An increase in the intestinal L/M ratio, but not the colonic permeability or sucralose excretion ratio, was found in UC patients in clinical remission compared with the findings in healthy controls [86]. Meanwhile, in both active CD and active extensive UC, the frequency of elevated intestinal permeability was significantly greater than in the inactive forms of both of these conditions, as measured using the L/R test [87]. Shaikh et al. investigated the membrane permeability in UC patients by administering a cocktail of sucrose/sucralose/mannitol/lactulose and measuring the urinary excretion of all sugars [88]. These indicators, with the exception of colonic permeability, showed increased membrane permeability compared with the findings in the historical control. The permeability to lactulose, rhamnose, and mannitol similarly did not differ among the three groups [89]. UC patients in remission did not show an effect of probiotic treatment or a change in their gut permeability, measured using a sugar test [89].

It has also been shown that the lactulose/mannitol ratio is associated with the later development of CD. Specifically, the membrane permeability of 1420 asymptomatic first-degree relatives of patients with CD was examined to observe whether they subsequently developed CD [90]. The results showed a higher membrane permeability in the group that did develop CD than in the group that did not. This suggests that increased membrane permeability is linked to pathogenesis.

The indicators of membrane permeability in IBD patients have not consistently shown increased membrane permeability in all studies. Although the reason for this has not been fully elucidated, one possible explanation is differences in the patient population. Another possible reason is the improved accuracy of the technology for analyzing sugar to provide more precise urinary excretion data. However, since high membrane permeability is associated with the onset of CD, there is a clear relationship between membrane permeability and IBD. Further in vivo studies of this issue will be conducted in conjunction with the results of in vitro studies.

## 6. Involvement of the Microbiome in Changes in Tight Junctions and Transporters

The causes of both diseases are not completely understood, but they are thought to be multifactorial diseases involving a genetic predisposition and environmental factors such as diet and a sanitary environment. With the recent development of genome-wide association studies (GWAS), nearly 200 disease-susceptibility genes have been identified in IBD [91,92]. As inferred from the functions of these disease-susceptibility genes, abnormalities in the regulation of innate and adaptive immune responses are thought to be involved in the pathology of IBD. In particular, the relationship between intestinal bacteria and the host immune system is attracting attention [93].

With regard to transporter expression, the involvement of the microbiome in p-glycoprotein expression has been suggested. Patients suffering from UC showed decreased p-glycoprotein expression in parallel with reductions in epithelium-derived anti-inflammatory endocannabinoids and luminal content (e.g., microbes or their metabolites), with a reduced ability to induce P-gp expression [94]. The importance of maintaining p-glycoprotein function is demonstrated by the findings that a lack of functional p-glycoprotein causes colonic inflammation and that single-nucleotide polymorphisms affecting the p-glycoprotein expression or function in humans are associated with IBD [95,96]. Furthermore, the development of spontaneous colitis with characteristics resembling those of human UC was observed in P-gp (mdr1a)-knockout mice [97,98,99].

Several non-clinical studies have revealed the healing of experimental colitis through the recovery of TJ proteins in parallel with the improved balance of the microbiota via the administration of sinapic acid and inosine [100,101]. In Caco-2 cells, *Lactobacillus rhamnosus* CY12, a favorable Gram-positive bacterial strain, was shown to ameliorate the loss of tight junctions through lipopolysaccharide treatment by improving the expression of the tight junction proteins claudin, ZO-1, and occludin [102]. 

Epidemiological studies have identified that a vitamin D deficiency is very common among patients with IBD, and lower serum levels correlate with higher disease activity [103]. It has been considered that an excessive immune response by CD4-positive helper T (Th) cells, especially Th1 and Th17 cells that secrete IFN-γ and IL-17, contributes to the chronic inflammation of IBD [104]. Many studies have now shown that vitamin D, whose pharmacological actions are regulated by the vitamin D receptor (VDR), suppresses inflammation and strengthens the intestinal barrier through the direct suppression of B and T cell proliferation and the regulation of cytokines [105,106,107]. In clinical studies, a vitamin D deficiency caused the decreased expression of VDR, occludin, E-cadherin, and zonula occludens-1 in patients with UC [108], and the reduced expression of claudin 1, occludin, zonula occludens, and junctional adhesion molecules in patients with CD [109]. In vitro studies using the inflamed tissues of patients with UC have demonstrated that treatment with vitamin D upregulated claudin-1 and claudin-2 and downregulated claudin-4 and claudin-7 [110]. In the context of the microbiota, the distribution pattern of the fecal microbiota is influenced by vitamin D, higher levels of which are associated with increased levels of beneficial bacterial species and decreased levels of pathogenic bacteria [111,112].

Food ingredients such as carbohydrate, protein, and lipid nutrients and food additives have been shown to change the composition of the microbiota [113]. Therefore, several dietary strategies to treat IBD have been examined, considering the possible effect of the composition of the microbiota on the disease state [114]. Among them, Gibson et al. proposed a promising nutritional strategy that directly targets the gut microbiota [115]. This approach is an intake of a diet low in fermentable oligosaccharides, disaccharides, monosaccharides, and polyols (FODMAPs) to prevent increased intestinal permeability due to bacterial overgrowth. Still, rich information is not available on the effect of low FODMAPs on the microbiota and inflammatory markers or disease activity [116]. Although several studies have been conducted to show the effectiveness of a low-FODMAP diet in the management of IBD [117,118], consideration of the adverse effects and issues with the intake of this diet should be required [119,120]. 

In summary, the relationship between the expression of tight junctions and transporters and IBD is multifaceted and interconnected with various aspects of the disease, including genetics, inflammation, the drug response, and interactions with the gut microbiota, whose distribution pattern might be affected by food. Further investigations are required to elucidate the relationships between transporter expression and IBD to gain a better understanding of this disease and develop more targeted treatments.

## 7. Utilization of Gut-on-a-Chip for Pathological Evaluation of IBD

Although the pathogenesis of IBD has not been completely elucidated, over 200 disease-associated genes have now been reported based on GWAS, and a genetic predisposition has been identified [91,92]. Many of the reported genes are related to intestinal immune abnormalities, epithelial barrier function abnormalities, mucosal secretion, and the secretion of antibacterial substances. In addition, the human intestinal tract is estimated to have more than 1000 species and hundreds of trillions of microorganisms, and to contain more than 100 times as many genes as the human genome. The metabolites derived from intestinal microorganisms play a role in regulating the intestinal environment by transducing intracellular signals, regulating the immune system, and suppressing harmful microorganisms. The progress of next-generation sequencing in recent years has enabled us to obtain an overview of the intestinal flora and has revealed the involvement of intestinal bacteria in the pathology of IBD. However, no evidence has emerged that an increase or decrease in specific intestinal bacteria causes IBD. This is despite fecal microbiota transplantation from a healthy donor being considered as a promising option to treat ulcerative colitis [121,122,123], and it being speculated that an imbalance of various bacterial species is significantly involved in this disease’s pathology. Against this background, it is essential to develop an in vitro IBD model that can recapitulate the contributing factors to the maximum extent possible and reconstruct the structure and microenvironment of the intestine [124,125]. However, it has been challenging to develop a novel in vitro gut model to mimic interactions with the microbiota [126]. One example is a co-culture model of colorectal-cancer-derived epithelial cells with a model probiotic/synbiotic regimen against colorectal cancer [127]. The superiority of a synbiotic regimen compared with individual prebiotic or probiotic treatments has been successfully demonstrated, with the former being shown to lead to the downregulation of genes involved in procarcinogenic pathways and drug resistance, and to a reduction in the oncometabolite lactate level. Recently, Yoon et al. used gut-on-a-chip to culture IBD patient cells with and without a peptide hydrogel treatment to validate the synergistic actions of the peptides and hydrogels used to treat IBD [128]. The data showed that the peptide hydrogel treatment for 96 h induced the significant structural recovery of IBD patient-derived cells in gut-on-a-chip, in parallel with an improvement in villus formation and ZO-1 expression. This is a new method for examining IBD based on the interaction between the microbiota and IBD. There are significant variations in the intestinal microbiota among IBD patients. It was shown that IBD patients exhibit relatively low intestinal bacterial diversity, with a particular loss of anaerobic bacteria [129]. An in vitro gut model enabling an evaluation of the interaction with the microbiota would help to reveal an appropriate regimen of fecal microbiota transplantation. However, it remains unclear whether dysbiosis precedes disease development or is a by-product of the disease [123]. The development of a novel in vitro gut model should provide an understanding of the involvement of the microbiota in IBD and insights for treating this disease [130].

## 8. Conclusions

This paper comprehensively summarizes the knowledge on the changes in membrane function in IBD, while also revealing that many issues remain unresolved. There are still many points left to be clarified regarding the findings presented here. For example, regarding IBD management through a low-FODMAP intake, it is thought that new knowledge can be obtained by conducting a membrane integrity evaluation using the subject’s symptoms and a matched pair of gastrointestinal tissue. In other words, the combination of in vivo symptoms and an in vitro functional evaluation will lead to the further understanding of pathological conditions and disease management. We hope that the presented information will lead to the development of experimental systems to shed light on IBD and also on drugs to treat this disease.

## Data Availability

Not applicable.

## Abbreviations

5-ASA	5-aminosalicylic acid
BCRP	breast cancer resistance protein
CD	Crohn’s disease
CYP	cytochrome P450
EGC	enteric glial cells
FITC	fluoresceinisothiocyanate
FODMAP	fermentable oligosaccharides, disaccharides, monosaccharides, and polyols
GWAS	genome-wide association studies
IBD	inflammatory bowel diseases
IBS	irritable bowel syndrome
Isc	short-circuit current
L/R	lactulose/L-rhamnose
L/M	lactulose/mannitol
MCT1	monocarboxylate transporter 1
MRP	multidrug resistance-associated protein
OATP	organic anion-transporting polypeptide
PD	potential difference
PepT	peptide transporter
TEER	transepithelial electrical resistance
TJ	tight junction
UGT	uridine 5′-diphospho-glucuronosyltransferase
UC	ulcerative colitis
VDR	vitamin D receptor
ZO	zonula and occuludens
